# Taste Preferences in Broilers: Effect of Age, Delivery Matrix, and Number of Chickens per Pen on Selection and Consumption Behaviour

**DOI:** 10.3390/ani14101507

**Published:** 2024-05-20

**Authors:** Paloma Cordero, Sofía Herrera-Alcaíno, Victoria Philp, Geraldine Muñoz, Daniela Luna, Sergio A. Guzmán-Pino

**Affiliations:** 1 Programa de Doctorado en Ciencias Silvoagropecuarias y Veterinarias, Campus Sur, Universidad de Chile, Santiago 8820808, Chile; paloma.cordero@veterinaria.uchile.cl (P.C.); sofia.herrera.a@ug.uchile.cl (S.H.-A.); 2 Departamento de Fomento de la Producción Animal, Facultad de Ciencias Veterinarias y Pecuarias, Universidad de Chile, Santiago 8820808, Chile; victoria.philp@veterinaria.uchile.cl (V.P.); geraldine.munoz@ug.uchile.cl (G.M.); daniela.luna.f@uchile.cl (D.L.)

**Keywords:** broiler chicken, chicken age, chicken number, compound matrix, consumption behaviour, taste preference

## Abstract

**Simple Summary:**

Taste perception in birds still needs to be clarified, and it is essential to advance investigations into improving nutrition in poultry farming. This study evaluated the effects of age, compound delivery matrix, and number of birds per pen on broilers’ preferences and consumption behaviour and obtained preference values for four taste compounds. The results indicated that chickens in the initial stage showed higher preferences and expressed more consumption behaviours. Birds in front of a water matrix showed higher preferences and increased their consumption behaviours, while in front of a ground wheat matrix, they increased their number of pecks. Furthermore, pairs of birds showed higher preference and consumption behaviours than single chickens. Finally, we registered significant preference values for sucrose and monosodium glutamate at different times. We concluded that the variables evaluated in this research condition the preferences and consumption behaviour of the birds, suggesting the use of early-stage chickens in pairs or groups and employing liquid matrices for compound delivery to enhance future trials.

**Abstract:**

Due to substantial differences between studies, the understanding of avian taste perception remains incomplete. Also, studies on chicken taste preferences have mainly focused on measuring consumption differences, neglecting consumption behaviour patterns. This study investigated how age, the compound delivery matrix, and the number of birds per pen affect broiler chicken preferences and consumption behaviour, and established their preference values for four taste compounds. Ninety-six one-day-old male broiler chickens (Ross 308) were divided into two age groups (initial: days 7–23; final: days 26–42), with two compound delivery matrices (water or ground wheat) and two numbers of birds (one or two chickens per pen), following a 2 × 2 × 2 factorial design. Four taste compounds (sucrose, monosodium glutamate (MSG), L-lysine, and calcium carbonate) were tested at different concentrations. Preferences were assessed at 2, 4, and 8 h post-test, along with recording various behavioural parameters. Initial-stage birds showed higher (*p* < 0.001) preference values, time of approach (TA), number of bouts (NB), duration of bouts (DB), and number of pecks (NP) than final-stage birds. Birds exposed to a water matrix also exhibited higher (*p* < 0.001) preference and NB, while those exposed to a ground wheat matrix showed a higher (*p* < 0.001) NP. Pens with a pair of birds had a higher (*p* < 0.003) 2 h preference, TA, NB, DB, and NP, than pens with a single chicken. Chickens showed significant preference values for 100 mM sucrose at 2 h (*p* = 0.025), 150 mM MSG at 4 h (*p* = 0.026) and 8 h (*p* = 0.013), and 300 mM MSG at 2 h (*p* = 0.013). We concluded that all the variables evaluated influence broilers’ taste preferences and consumption behaviour during selection tests. Future studies should prioritize including chickens in the initial stage of the production cycle, testing them in pairs or groups, and delivering compounds via a liquid matrix.

## 1. Introduction

The sense of taste is part of animals’ chemosensory system that links chemical compounds with pleasant or unpleasant stimuli, conditioning feeding behaviour by stimulating or discouraging food consumption [1]. Taste buds are specialized sensory cells that detect tastes associated with food nutrients [2]. In broiler birds, it has been established that there are 507 taste buds at the level of the palate and 260 at the base of the oral cavity [3], which provide them with the ability to detect five basic tastes in nature. These fundamental tastes include: umami taste, associated with the perception of protein digestion products; salty, associated with the detection of electrolytes; sour and bitter, associated with the detection of potentially toxic or spoiled substances; and fatty, associated with the detection of lipid digestion products [4,5]. The evolution of this system in living beings has allowed for the identification of foods according to their nutritional quality [6], this being the paramount importance of the sense of taste as a potential tool to achieve improvements in nutrition topics in broiler birds [7].

Taste perception in birds has yet to be fully elucidated due to the significant variability among studies. The first taste preference tests in birds date back to the 1950s, when it was suggested that preferences for a compound may vary by factors specific to an organism, such as its nutritional requirements [8,9,10], by acquired learning [8] or by the taste of a food [8,11]. It has been reported that preferences were expressed within a specific concentration range according to compound, inferring that, in addition to taste, concentration would be a determinant of choice in this type of test in birds [12]. Other studies found that preferences for a compound may also be conditioned by organism-specific factors such as the age of exposure of birds at the time of preference testing [13,14,15] and by external factors such as the delivery matrix of taste compounds [16], and the animal density effect given by the number of birds exposed to a test determining a social component [17]. Taste preferences and aversions in birds have been analyzed in numerous studies where different compounds have been tested, such as umami, calcium, and salty taste representatives [16]; bitter, sweet, and umami [18]; and umami, salty, and sour [19]. Several of these tests have taken into account the type of compound delivery matrix, the age of the birds, and the number of birds for testing, which has determined a high variability in the results obtained. In addition, studies on taste preferences in birds have been based exclusively on differential consumption measurements without considering feeding behaviour patterns. Therefore, research incorporating all the above-mentioned variables into a single analysis would determine the best model for evaluating taste preference tests. Moreover, published studies have not considered animal–environment interactions, observed through behavioural analysis, which would determine better resource utilization efficiency and animal health [20]. The objectives of the present work were (i) to evaluate the effect of age, compound delivery matrix, and number of birds per pen on gustatory preferences and the consumption behaviour of broiler chickens, and (ii) to determine the preference values of broiler chickens for four different sapid compounds.

## 2. Materials and Methods

### 2.1. Animals, Housing, and Diets

The field trial was developed at the Experimental Unit for Poultry Nutrition and Production of the Faculty of Veterinary and Animal Sciences (FAVET) of the University of Chile (UCH). This facility has a conventional structure with natural ventilation and 32 floor pens with wood-shaving beds. The animals used were sourced from a commercial poultry company in the Metropolitan Region of Chile. Ninety-six one-day-old male broiler chickens (Ross 308) were used in two experimental flocks with 48 chickens each for 42 days. The experimental flocks were examined sequentially, with a 7-day rest period for cleaning, disinfection, and sanitary vacuuming. The feeding program (Supplementary Table S1) consisted of starter and grower commercial diets formulated to meet or exceed all nutrient requirements set by the NRC (1994) and the guidelines set by the breeder [21] as previously described [22]. Each diet was offered ad libitum to chickens from 1 to 23 and 24 to 42 days, respectively. Water was also provided ad libitum until the start of the preference tests, and the dark and light cycles followed the breeder’s guidelines [23].

### 2.2. Experimental Design

**Figure 1:**
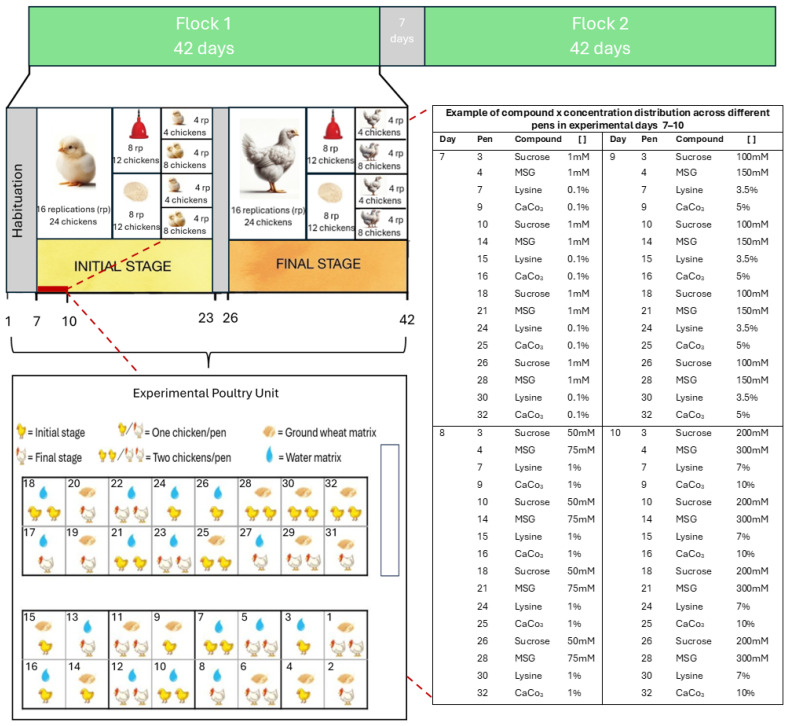
Schematic representation of the experimental design used in the study.

In each experimental flock, one-day-old birds were weighed for homogeneous distribution. The distribution of the animals within the 32 pens consisted of 16 pens with one bird and 16 pens with a pair of birds. Birds were subjected to an initial phase of 7 days of acclimatization to the environmental conditions of the Poultry Unit prior to the start of the tests. Animals that perished within the first seven days of life were replaced prior to the commencement of the tests. Nonetheless, the rate of mortality recorded was low and below the expected standards. On day 7, the preference tests were started. For this purpose, pens were assigned following a 2 × 2 × 2 factorial design that considered two bird age groups (initial, chickens between days 7 and 23; final, chickens between days 26 and 42), two compound delivery matrices (water or ground wheat), and two numbers of animals per pen (one chicken or two chickens). Four sapid compounds were evaluated at four different concentrations: sucrose (sweet taste representative) at 1, 50, 100, and 200 mM; monosodium glutamate (MSG, umami taste representative) at 1, 75, 150, and 300 mM (Prinal S.A., Santiago, Chile); L-lysine (umami taste representative) at 0.1, 1, 3.5, and 7% (Veterquímica S.A., Santiago, Chile) and calcium carbonate (CaCO_3_, calcium taste representative) at 0.1, 1, 5, and 10% (Proa S.A., Santiago, Chile). This configuration resulted in 16 pens for the initial-stage bird preference analysis conducted on days 7–23 of the cycle, and 16 for the final-stage bird preference analysis conducted on days 26–42. Within the 16 pens from both initial- and final-stage birds, eight evaluated preferences under a water matrix and eight evaluated preferences under a ground wheat matrix (Figure 1). Delivery was rotated throughout the 16 days, determining that in each pen, all compounds were tested in all concentrations. The matrices arranged in the pens consisted of two identical drinkers or feeders positioned 20 cm apart, following the methodology of previous studies [12,16,18]. One of them contained drinking water or ground wheat (neutral option, “N”), and the other was a sapid compound diluted in water or ground wheat at a specific concentration (compound × concentration, “C × []”).

### 2.3. Taste Preference Analysis

The preference tests lasted 8 h, starting at 09:00 a.m. until 5:00 p.m., with a previous fasting hour at 08:00 a.m. During this period, access to the ad libitum delivery feeders or drinkers was restricted within each pen, depending on the type of matrix used for the preference test. During the 16 days of testing of initial- and final-stage birds, all compound × concentration combinations were tested. The compound delivery order among the different pens was counterbalanced, while the tested concentrations of each compound were offered in increasing order. The average consumption of each drinker or feeder was estimated by weight loss of the matrices through the subtraction between the amount delivered and the amount withdrawn at 2, 4, and 8 h after the start of the test.

Given that the age of the birds is estimated based their body weight (BW), birds’ consumption was deduced by their metabolic weight for each day of testing. This value was obtained to equal the differential consumption capacities associated with the age of the birds. It was expressed in g/kg of BW, which was used to calculate the preference for each compound. The preference value was calculated as the percentual consumption of C × [] concerning total intake (C × [] consumption plus N consumption) and was compared with the neutral value of 50% as previously described [24,25].

### 2.4. Consumption Behaviour Analysis

A 4K sports camera (1080 megapixels, 720 fps; Microlab^®^, Santiago, Chile) was placed at the top of each pen to record both matrices offered during the preference tests and bird activity inside the pens. A total of 20 min of video recording per day was recorded, consisting of 10 min at the beginning of the trial immediately after the matrices were offered (09:00 a.m.) and 10 min at the end of the trials, one hour before finishing them (4:00 p.m.). The parameters considered for the analysis were based on the study by Shynkaruk et al. [26]:

- Time of approach (TA): The period in which a bird was at the edge observing the matrix. It began when the animal approached the source offered for consumption and ended when it turned around, walked away, or lay down;
- Number of bouts (NB): The number of bouts recorded within the TA;
- Duration of bouts (DB): Active consumption with no break of more than 10 s between pecks;
- Number of pecks (NP): The number of pecks made by a bird to the matrix offered. Regarding the consumption of solid sources, it was observed as a hammer-like head downward and back upward movement. Regarding the consumption of liquid sources, it was observed as a downward head movement and upward glance and then back to its original position.

### 2.5. Statistical Analysis

The variables examined, namely preference at 2 h, 4 h, 8 h, time of approach, number of bouts, duration of bouts, and number of pecks, were analyzed with three-way ANOVA using the GLM procedure of SAS (version 9.4, SAS Institute; Cary, ND, USA). The statistical model considered the factors of birds’ age (initial or final stage), compound delivery matrix (water or ground wheat), number of chickens per pen (1 or 2 chickens), and all their possible interactions (age × delivery matrix, age × chicken number, delivery matrix × chicken number, and age × delivery matrix × chicken number). Shapiro–Wilk and Levene’s tests for normality and homogeneity of variance were performed on each variable before ANOVA. LSMeans compared mean values with Tukey’s post hoc test. In addition, the mean preference values for the four sapid compounds analyzed were compared with the neutral value of preference (50%) by Student’s *t*-test using the MEANS procedure of SAS. For all analyses, a significance level α of 0.050 was considered, and values of 0.050 < *p* < 0.100 were considered as a tendency to significance.

## 3. Results

### 3.1. Age

**Figure 2:**
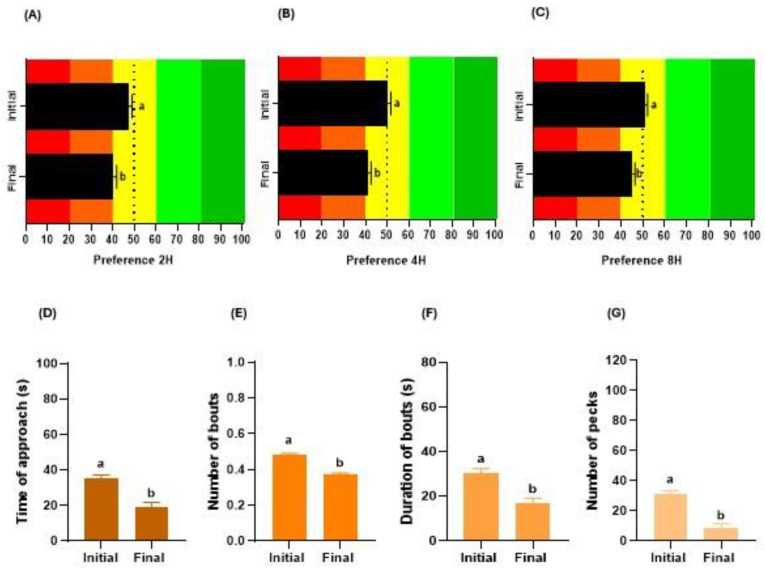
Effect of bird age (initial, chickens between days 7 and 23; final, chickens between days 26 and 42) on their gustatory preferences and consumption behaviour. Influence on the preference (%) of broilers at 2 h (**A**), 4 h (**B**), or 8 h (**C**) of testing. The red and orange colors in the graphs represent the aversion zone, the yellow color the indifference zone, and the green colors the preference zone. Impact on the time of approach (**D**), number of bouts (**E**), duration of bouts (**F**), and number of pecks (**G**) during choice tests. In each graph, different letters (a, b) denote significant differences (*p* ≤ 0.050).

The age factor influenced the gustatory preferences of broiler chickens. Initial-stage birds (7–23 days) showed higher preference values than final-stage birds (26–42 days) at 2 (*p* < 0.001; Figure 2A), 4 (*p* < 0.001; Figure 2B), and 8 h (*p* < 0.001; Figure 2C) of testing. Concerning consumption behaviour, age also affected the parameters evaluated. Initial-stage birds showed a higher TA (*p* < 0.001; Figure 2D), NB (*p* < 0.001; Figure 2E), DB (*p* < 0.001; Figure 2F), and NP (*p* < 0.001; Figure 2G) than final-stage birds.

### 3.2. Delivery Matrix

**Figure 3:**
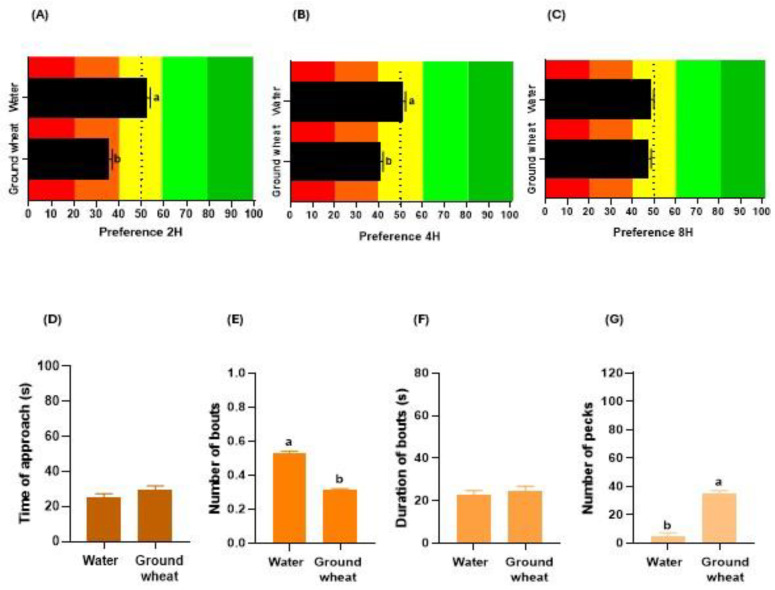
Effect of compound delivery matrix (water or ground wheat) on the gustatory preferences and consumption behaviour of broiler chickens. Influence on the preference (%) of birds at 2 h (**A**), 4 h (**B**), or 8 h (**C**) of testing. The red and orange colors in the graphs represent the aversion zone, the yellow color the indifference zone, and the green colors the preference zone. Impact on the time of approach (**D**), number of bouts (**E**), duration of bouts (**F**), and number of pecks (**G**) during choice tests. In each graph, different letters (a, b) denote significant differences (*p* ≤ 0.050).

**Figure 4:**
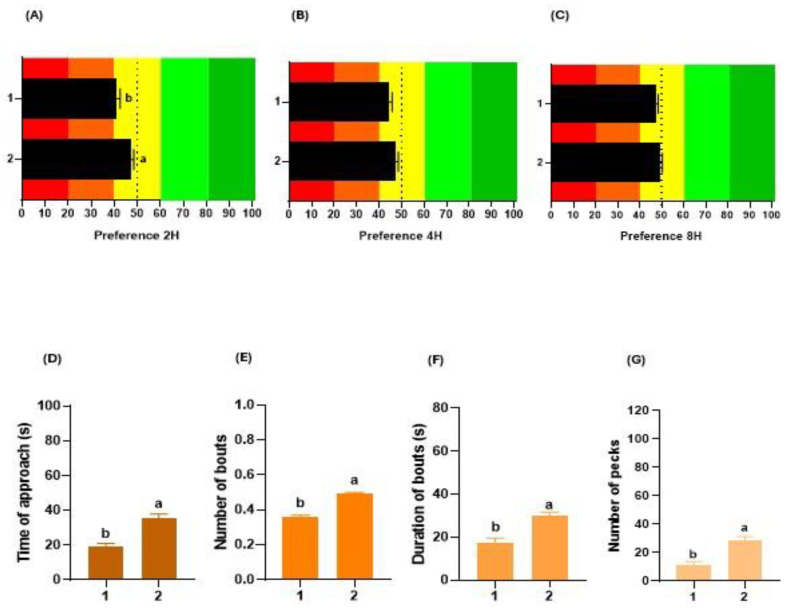
Effect of the number of chickens per pen (1 or 2 chickens) on their gustatory preferences and consumption behaviour. Influence on the preference (%) of broilers at 2 h (**A**), 4 h (**B**), or 8 h (**C**) of testing. The red and orange colors in the graphs represent the aversion zone, the yellow color the indifference zone, and the green colors the preference zone. Impact on the time of approach (**D**), number of bouts (**E**), duration of bouts (**F**), and number of pecks (**G**) during choice tests. In each graph, different letters (a, b) denote significant differences (*p* ≤ 0.050).

The factor delivery matrix of the compounds impacted the gustatory preferences of broiler chickens. Birds showed a higher preference for the water matrix at 2 h (*p* < 0.001; Figure 3A) and 4 h (*p* < 0.001; Figure 3B), while there was no difference in preference from both matrices at 8 h (*p* = 0.401; Figure 3C). Regarding consumption behaviour, the delivery matrix did not affect the TA (*p* = 0.125; Figure 3D) nor the DB (*p* = 0.475; Figure 3F). In contrast, a higher NB (*p* < 0.001; Figure 3E) was observed in birds exposed to a water matrix, and a higher NP (*p* < 0.001; Figure 3G) in birds exposed to a ground wheat matrix.

### 3.3. Number of Chickens

The number of chickens per pen affected the taste preferences of broiler chickens with a higher value in pens with a pair of birds at 2 h (*p* < 0.003; Figure 4A), which was not observed at 4 h (*p* = 0.111; Figure 4B) or 8 h of testing (*p* = 0.189; Figure 4C). Concerning consumption behaviour, the number of birds that performed preference tests affected all the parameters evaluated. Pens with a pair of chickens showed a higher TA (*p* < 0.001; Figure 4D), NB (*p* < 0.001; Figure 4E), DB (*p* < 0.001; Figure 4F) and NP (*p* < 0.001; Figure 4G) than pens with a single chicken.

### 3.4. Age × Matrix × Chickens

**Figure 5:**
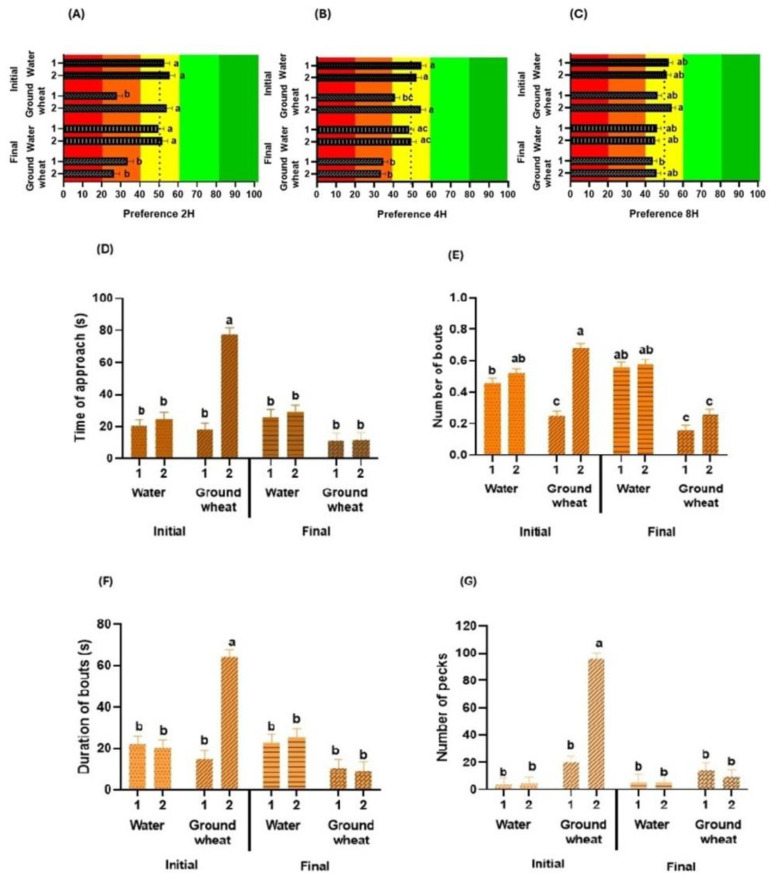
Effect of the interaction between bird age (initial, chickens between days 7 and 23; final, chickens between days 26 and 42), compound delivery matrix (water or ground wheat), and number of chickens per pen (1 or 2 chickens) on their gustatory preferences and consumption behaviour. Influence on the preference (%) of broilers at 2 h (**A**), 4 h (**B**), or 8 h (**C**) of testing. The red and orange colors in the graphs represent the aversion zone, the yellow color the indifference zone, and the green colors the preference zone. Impact on the time of approach (**D**), number of bouts (**E**), duration of bouts (**F**), and number of pecks (**G**) during choice tests. In each graph, different letters (a, b, c) denote significant differences (*p* ≤ 0.050).

The interaction between the factors of age, matrix, and chickens influenced the gustatory preferences of broiler chickens across the different measurements. The preferences at 2 h (in percentages of preferences) of initial-stage and water matrix birds in pens with one chicken or a pair of chickens were similar to those of initial-stage and ground wheat matrix birds with a pair of chickens (*p* = 1.000) but were higher than pens with one chicken (*p* < 0.001; Figure 5A). Preferences for final-stage birds and water matrix in pens with one chicken or a pair of chickens were higher than those of final-stage birds and ground wheat matrix in pens with one chicken and a pair of chickens (*p* = 0.002). The water matrix had a more significant impact on preference values in final-stage birds (*p* < 0.001) than in initial-stage birds (*p* = 1.000). The number of chickens determined a higher taste preference in pens with two chickens compared to one chicken during their initial stage (*p* < 0.001) as compared to birds in the final stage (*p* = 0.612).

At 4 h of testing, the preferences of initial-stage and water matrix birds in pens with one or two chickens were similar to those of initial-stage, ground wheat matrix birds with a pair of chickens (*p* = 0.998), but were higher than those in pens with only one chicken (*p* < 0.025; Figure 5B). Preferences for final-stage birds and water matrix in pens with one or two chickens were higher than those of final-stage birds and ground wheat matrix with one or two chickens (*p* = 0.003). Delivery matrix had a higher incidence in final-stage birds (*p* = 0.002) than in initial-stage birds (*p* = 0.998). Preference according to the number of chicks per pen had a more significant influence in initial-stage birds (*p* = 0.002) than in final-stage birds (*p* = 0.001).

The preference values at 8h were not affected by the variable’s delivery matrix and number of chickens per pen in both initial- (*p* = 0.158) and final-stage birds (*p* = 0.993; Figure 5C). The pairs of initial-stage chickens with a ground wheat matrix showed a higher preference than pens with a final-stage chicken with the same matrix (*p* = 0.011).

Relative to consumption behaviour, the TA of pens with a pair of initial-stage chickens and a ground wheat matrix was significantly higher than that of the rest of the birds under the different combinations of factors (*p* < 0.001; Figure 5D). Similarly, the NB in initial-stage birds and solid matrix was higher in pens with a pair of chickens relative to pens with only one chicken (*p* < 0.001; Figure 5E), and pens with only one chicken and water matrix (*p* < 0.001). In final-stage birds, the water matrix resulted in a higher NB in pens with one (*p* < 0.001) and two chickens (*p* < 0.001) compared to the ground wheat matrix. The number of chickens did not significantly influence the NB of final-stage birds without considering the delivery matrix (*p* = 0.999). Finally, pens with a pair of initial-stage chickens and a solid matrix had a higher DB (*p* < 0.001; Figure 5F) and NP (*p* < 0.001; Figure 5G) than the rest of the birds under the different combinations of factors evaluated.

### 3.5. Broilers Preference for Sapid Compounds

Considering the previously presented results, the combination of initial-stage chickens provided a water matrix and housed in pairs per pen was selected to study the chickens’ preference for compounds representative of sweet, umami, and calcium tastes. The results are shown in Table 1. For sucrose concentrations of 100 mM, a significant preference was observed at 2 h (*p* = 0.025), which then showed a tendency to be preferred at 4 h (*p* = 0.088). In the case of MSG at a concentration of 150 mM, a tendency to preference was observed at 2 h (*p* = 0.077), which reached significant preference at 4 h (*p* = 0.026) and 8 h (*p* = 0.013). On the other hand, for MSG at a concentration of 300 mM, a significant preference was observed only at 2 h (*p* = 0.013); however, this preference was not sustained in the hours following evaluation. For L-lysine at a concentration of 3.5%, a preferential tendency was observed only at 4 h of evaluation (*p* = 0.099). Regarding CaCO_3_, no significant preference was observed under any of the evaluated conditions.

## 4. Discussion

The present study evaluated the gustatory preferences and consumption behaviour of broiler chickens for four sapid compounds (sucrose, MSG, L-lysine, and CaCo_3_) offered through choice tests, incorporating the following variables: bird age (initial stage, 7–23 days vs. final stage, 26–42 days), compound delivery matrix (water vs. ground wheat), number of birds per pen (one vs. two chickens), and their interaction. Preference values were obtained by analyzing the percentage consumption of the matrices that included the taste-active compounds at 2, 4, and 8 h of delivery. On the other hand, consumption behaviour was evaluated by video recording analysis of quantifying patterns associated with the ingestion of the delivered compounds under a previously published model [26].

In this work, we observed higher preference values in initial-stage birds compared to final-stage birds at 2, 4, and 8 h after the delivery of compounds. These results are consistent with a previously published study by our group, which determined a higher expression of taste receptors in the oral cavity of birds at day 7 of the cycle compared to birds at day 26, inferring and enhanced ability for oral nutrient detection linked to the increased taste sensitivity of the birds, and therefore a higher intake of compounds [22]. Also, we determined a significant effect of age on the consumption behaviour, affecting the time of approach, number of bouts, duration of bouts, and number of pecks higher in birds in the initial stage, and demonstrating more significant feeding activity compared to final-stage birds. These results agree with the results from previous studies, such as Aldridge et al. [27], who determined that 14-day-old chickens visited the feeders and drinkers more frequently than 21- and 40-day-old chickens. Similarly, Shynkaruk et al. [26] indicated that as broiler age increased, fewer visits to the feeders were observed; however, time spent at the feeders increased, which determined a more significant number of bouts and longer intervals between bouts. Nonetheless, their study is not fully comparable to this one since the measurement times of the trials varied substantially, with 24 h behavioural recordings versus 20 min recordings per trial day, respectively, with no measurements of the intervals between bouts.

The delivery matrix factor impacted the taste preferences of the birds that were reflected in higher preferences for the water matrix at 2 and 4 h after the presentation of the compounds. This finding reinforces the recommendation to use liquid matrices for this type of test, incorporating the input of a higher selection of birds for a matrix that offers a high degree of simplicity, precision, and accuracy [18] to achieve an optimal execution of feeding trials. Regarding consumption behaviour, the matrix effect showed significance only for the number of bouts and number of pecks recorded, with more bouts in pens with a water matrix and more pecks in pens with a ground wheat matrix. The above can be explained by how birds consume water and feed, where pecking for feed is characterized by a hammering motion towards an offered source, where the chicken repeatedly lowers and raises its head. In contrast, in the case of water, the chicken lowers its head towards the source, then looks up and puts it back to its original position [26]. Therefore, this differential behavioural expression of consumptions would determine that the results obtained in the present study were expected for the variables analyzed. Regarding the existing literature, only Cheled-Shoval et al. [18] used water as the delivery matrix; thus, the findings of this research are valuable in determining the best alternative for the compound delivery matrix for the development of consumption behaviour tests. Other authors that have compared matrices for this type of testing evaluated the delivery of two solid matrices and observed higher bird preferences for ground wheat over corn starch [16]. Also, Iqbal et al. [19] noted low ground wheat consumption in birds that developed preference tests. Thus, no previous studies have compared the use of solid and liquid matrices in preference tests that could be comparable to the results of this study.

Broiler preferences were conditioned to the number of birds in each pen. Specifically, at 2 h after the delivery of the compounds, higher-preference values were observed in pens with a pair of chickens compared to pens with a single chicken. These findings are discordant with the study of Iqbal et al. [19], who analyzed this variable and determined that the number of animals did not influence consumption. However, we determined that the consumption behaviour of the birds was also influenced by the number of animals, which consolidates the results of preferences, given that there was increased activity reflected in a higher time of approach, the number of bouts, duration of bouts, and number of pecks in pens with two chickens compared to pens with only one chicken. It should be noted that Collins and Sumpter [17] mentioned the effect of social facilitation in chickens, positing that there is an incentive for developing an activity in the presence of more animals exercising that activity. Furthermore, Li et al. [20] showed that the probability of a chicken visiting the feeder increases when more animals consume feed, which is consistent with our results. Such premises may explain the effects observed in this research.

Based on the results obtained on the effect of the previously discussed variables, we analyzed chickens’ preferences for four taste-active compounds following an evaluation model with a pair of birds between 7 and 23 days of age, offered liquid matrices. The selections revealed interesting results, especially for the sweet and umami representative compounds. Chickens, unlike other animals, do not express the T1R2 gene [28], which translates into a low sensitivity to sweet taste, reflected in their indifference to glucose and sucrose solutions [18,29]. Notably, despite the absesnce of the gene coding for the mammalian sweet taste receptor subunit in chickens, which implies the T1R2/T1R3 pathway is inactive [30], our data indicate that chickens could differentiate and significantly prefer 100 mM sucrose solutions after 2 h of exposure. This may suggest an alternative gustatory mechanism that enables them to recognize and respond to this concentration, which could be helpful in the development of a feeding strategy that seeks to increase the initial intake without negatively affecting long-term preferences. This is because the rejection of high-sucrose-concentration solutions previously reported in the literature [18] suggests a T1R-independent sweet taste sensory mechanism, possibly linked to glucose transporters such as SGLT1 and disaccharide digestive enzymes [31,32]. The information above highlights the need for further research on this phenomenon to fully utilize its applications in poultry nutrition.

Regarding the umami taste representative compounds, the presence of amino acid sensors responsible for its perception, such as the T1R1/T1R3 heterodimer, the metabotropic glutamate receptors mGluR1 and mGluR4 [33,34,35], and the GPR92 receptor [22], is recognized. Although there is evidence of gustative perception of the umami stimuli in broiler chickens [36], their null preference for MSG plus inosine monophosphate solutions in two-bottle choice tests [18,29] highlights the differences in sensitivity between species and the complexity of their analysis in birds. In our screening, particularly in response to high concentrations of MSG, we observed a significant preference for a 300 mM solution at 2 h that did not persist over time. This finding could imply a short-term perception threshold response to high MSG concentrations, a phenomenon that may be influenced by sensory fatigue or taste adaptation over time. In contrast, the 150 mM MSG solution tended to be preferred at 2 h, reaching significant preference at 4 and 8 h. The differential selection pattern indicates possible post-ingestive effects that may influence feed discrimination. The delayed preference suggests that birds may be experiencing positive consequences after digestion and absorption of the MSG solution, resulting in a learned association that favours the selection of this concentration at later times. These divergent behaviours in temporal preference highlight the complexity of the taste sensory system and possible post-ingestive effects in broiler chickens.

## 5. Conclusions

The variables assessed in this research, specifically age, delivery matrix, and number of birds per pen, influence the taste preferences of broiler chickens during selection tests. Initial-stage birds (7–23 days) showed higher levels of preference than final-stage birds (26–42 days). Similarly, we observed higher preferences when broilers were offered water matrices instead of ground wheat. Additionally, pens with two chickens exhibited higher mean preference values compared to pens with a single bird. Consumption behaviour was also affected by the variables given above, showing changes in the time of approach, number of bouts, duration of bouts, and number of pecks with the offered sources. We determined that broilers showed significant preferences for 100 mM sucrose solutions after 2 h of evaluation, 150 mM MSG at 4 and 8 h, and 300 mM MSG after 2 h of compound offerings. The findings of this study provide valuable information about the feeding behaviour of broiler chickens during taste preference tests that can be used as a tool to improve the design of feeding trials. In future field studies, it is essential to consider the execution of tests that include the use of chickens in the initial stage of the productive cycle, in pairs or groups of birds, and the delivery of compounds in a liquid matrix, to contribute to the improvement of nutritional strategies that promote sustainable feeding in poultry farming.

## Figures and Tables

**Table 1:** Preference values for different concentrations of sucrose, MSG, L-lysine, and CaCO_3_ solutions in broiler chickens at 2 h, 4 h, and 8 h of selected test ^1^.

Compound	Concentration	2 h			4 h			8 h		
		Preference (%)	SEM	*p*-Value	Preference (%)	SEM	*p*-Value	Preference (%)	SEM	*p*-Value
Sucrose	1 mM	58.9	8.0	0.306	56.6	5.4	0.259	55.3	5.0	0.323
	50 mM	40.6	7.7	0.259	40.7	5.5	0.132	43.9	5.2	0.284
	100 mM	66.8 *	5.9	0.025	61.0	5.6	0.088	55.0	5.1	0.357
	200 mM	49.9	6.1	0.995	47.3	4.3	0.549	47.9	5.7	0.723
MSG	1 mM	60.7	5.2	0.080	53.4	3.9	0.418	51.1	4.5	0.817
	75 mM	50.1	5.5	0.991	48.3	4.3	0.696	49.5	4.3	0.913
	150 mM	62.3	5.9	0.077	61.9 *	4.2	0.026	62.2 *	3.7	0.013
	300 mM	66.4 *	4.9	0.013	59.2	5.3	0.128	45.8	4.4	0.369
L-lysine	0.1%	58.9	6.1	0.181	49.9	7.9	0.994	55.8	4.1	0.202
	1%	51.7	12.6	0.898	43.9	7.1	0.421	47.6	5.2	0.652
	3.5%	53.5	6.5	0.608	63.9	7.3	0.099	56.2	3.9	0.154
	7%	46.6	8.9	0.711	43.6	6.4	0.354	39.9	6.5	0.165
CaCO_3_	0.1%	50.8	5.6	0.898	50.9	4.6	0.854	53.3	3.5	0.375
	1%	55.6	9.4	0.573	53.8	6.3	0.571	53.4	5.7	0.573
	5%	55.2	6.3	0.438	49.8	5.5	0.967	52.0	3.5	0.588
	10%	63.1	9.7	0.219	51.7	10.5	0.874	55.2	6.9	0.472

^1^ A pair of birds between 7 and 23 days of age, offered a water matrix, performed the tests. Asterisks (*) denote significant preference values (*p* ≤ 0.050).

## Data Availability

The data are available upon reasonable request to the submitting author.

## Supplementary Materials

The following supporting information can be downloaded at: https://www.mdpi.com/article/10.3390/ani14101507/s1, Table S1: Composition and chemical analysis of the starter and grower diets used in the experiment.
