# Supplementary material for: Taste Preferences in Broilers: Effect of Age, Delivery Matrix, and Number of Chickens per Pen on Selection and Consumption Behaviour
Source: Animals (Basel). 2024 May 20;14(10):1507. doi: 10.3390/ani14101507 (PMC11117319; doi:10.3390/ani14101507)
Supplement: Supplementary file 1 [file animals-14-01507-s001.zip › applsci-2982602-Supplementary.pdf]

animals MDPI

Supplementary Table S1. Composition and chemical analysis of the starter and grower diets used in the experiment.

Item	Starter	Grower
Ingredients (g/kg)		
Corn	554.2	630.9
Soybean meal (47% protein)	270.3	192.5
Ground wheat	50.0	50.0
Rapeseed meal	40.0	70.0
Gluten meal (60% protein)	30.0	0
Olein oil	16.5	20.3
$CaCO_3$	14.4	16.3
$CaHPO_4$	11.8	7.1
NaCl	4.4	4.1
Lys	2.2	1.6
Met	2.1	3.2
Micofix plus[1]	0.005	0.05
Coccidiostat	0.5	0.5
Multivitamins-mineral-phytase[2]	2.0	2.0
Formicit dry[3]	1.0	1.0
Analyzed nutrient composition (%)		
Dry matter	88.9	88.7
Crude protein	22.9	18.1
Crude fiber	3.4	4.3
Ether extract	3.8	4.9
NNE	53.7	55.0
Ash	5.1	6.4

[1]Integral solution for mycotoxins (Virbac Centrovet, Santiago, Chile); [2]Contains per kilo of premix: 8000 UI of Vit. A, 2500 UI of Vit. D3, 15 UI of Vit. E, 1.5 mg of K3, 1.5 mg of Vit. B1, 5 mg of Vit. B2, 35 mg of niacin, 13.1 mg of calcium pantothenate, 2.49 mg of Vit. B6, 0.012 mg of Vit. B12, 1 mg of folic acid, 0.1 mg of biotin, 399.5 mg of choline, 25 mg of Fe, 70 mg of Mn, 60 mg of Zn, 6 mg of Cu, 0.15 mg of Se, 100 mg of antox, 0.5 mg of I,100 mg of Hostazym X, 50 mg of Optiphos G; [3]Preservative anti-salmonella sp. For food preservation (Veterquímica SA, Santiago, Chile).
